# A Mouse Model for Chikungunya: Young Age and Inefficient Type-I Interferon Signaling Are Risk Factors for Severe Disease

**DOI:** 10.1371/journal.ppat.0040029

**Published:** 2008-02-15

**Authors:** Thérèse Couderc, Fabrice Chrétien, Clémentine Schilte, Olivier Disson, Madly Brigitte, Florence Guivel-Benhassine, Yasmina Touret, Georges Barau, Nadège Cayet, Isabelle Schuffenecker, Philippe Desprès, Fernando Arenzana-Seisdedos, Alain Michault, Matthew L Albert, Marc Lecuit

**Affiliations:** 1 Groupe “Microorganismes et Barrières de l'Hôte”, Institut Pasteur, Paris, France; 2 Équipe Avenir INSERM U604 Paris, France; 3 Unité “Cellules Souches et Développement”, CNRS URA 2578, Institut Pasteur, Paris, France; 4 INSERM, U841, Team 10, Créteil, France; 5 AP-HP, Groupe Hospitalier Albert Chenevier-Henri Mondor, Département de Pathologie, Créteil, France; 6 Université Paris XII, Faculté de Médecine, Créteil, France; 7 Groupe “Immunobiologie des Cellules Dendritiques”, Institut Pasteur, Paris, France; 8 INSERM U818, Paris, France; 9 Unité “Interactions Bactéries-Cellules”, Institut Pasteur, Paris, France; 10 Département de Gynécologie et Obstétrique, Groupe Hospitalier Sud-Réunion, Saint-Pierre, La Réunion, France; 11 Plateforme de Microscopie Ultrastructurale, Institut Pasteur, Paris, France; 12 Centre National de Référence des Arbovirus, Institut Pasteur Lyon, France; 13 Unité “Interactions Moléculaires Flavivirus-Hôtes”, Institut Pasteur, Paris, France; 14 Laboratoire “Pathogénie Virale Moléculaire”, Institut Pasteur, Paris, France; 15 CNRS URA 3015, Paris, France; 16 Laboratoire de Microbiologie, Groupe Hospitalier Sud-Réunion, Saint-Pierre, Ile de la Réunion, France; 17 Centre d'Infectiologie Necker-Pasteur, Hôpital Necker-Enfants malades, Assistance Publique-Hôpitaux de Paris, Université Paris Descartes, Paris, France; The Scripps Research Institute, United States of America

## Abstract

Chikungunya virus (CHIKV) is a re-emerging arbovirus responsible for a massive outbreak currently afflicting the Indian Ocean region and India. Infection from CHIKV typically induces a mild disease in humans, characterized by fever, myalgia, arthralgia, and rash. Cases of severe CHIKV infection involving the central nervous system (CNS) have recently been described in neonates as well as in adults with underlying conditions. The pathophysiology of CHIKV infection and the basis for disease severity are unknown. To address these critical issues, we have developed an animal model of CHIKV infection. We show here that whereas wild type (WT) adult mice are resistant to CHIKV infection, WT mouse neonates are susceptible and neonatal disease severity is age-dependent. Adult mice with a partially (IFN-α/βR^+/−^) or totally (IFN-α/βR^−/−^) abrogated type-I IFN pathway develop a mild or severe infection, respectively. In mice with a mild infection, after a burst of viral replication in the liver, CHIKV primarily targets muscle, joint, and skin fibroblasts, a cell and tissue tropism similar to that observed in biopsy samples of CHIKV-infected humans. In case of severe infections, CHIKV also disseminates to other tissues including the CNS, where it specifically targets the choroid plexuses and the leptomeninges. Together, these data indicate that CHIKV-associated symptoms match viral tissue and cell tropisms, and demonstrate that the fibroblast is a predominant target cell of CHIKV. These data also identify the neonatal phase and inefficient type-I IFN signaling as risk factors for severe CHIKV-associated disease. The development of a permissive small animal model will expedite the testing of future vaccines and therapeutic candidates.

## Author Summary

Chikungunya virus (CHIKV) is transmitted by mosquito bites. CHIKV has recently re-emerged and is responsible for a massive outbreak in the Indian Ocean region and India. It has also reached Italy, indicating that CHIKV has a great potential to spread globally. Infection from CHIKV typically induces a mild disease in humans, characterized by a flu-like syndrome associated with muscle and joint pain and rash. Cases of severe infection involving the central nervous system (CNS) have recently been described, notably in neonates. We have developed the first animal model for CHIKV infection and studied the pathophysiology of the resulting disease. We show here that mouse neonates are susceptible to CHIKV and neonatal disease severity is age-dependent. Adult mice with a partial or complete defect in type-I interferon pathway develop a mild or severe infection, respectively. In mice with a mild infection, CHIKV primarily targets muscle, joint and skin fibroblasts, a cell and tissue tropism similar to that observed in biopsy samples of CHIKV-infected humans. In case of severe infections, CHIKV also disseminates to the CNS. Our work indicates that CHIKV-associated symptoms perfectly match viral tissue and cell tropisms, and demonstrate that the fibroblast is a prominent target cell of CHIKV. It also identifies the neonatal phase and inefficient type-I interferon signaling as risk factors for severe CHIKV-associated disease. The development of a permissive small animal model will expedite the testing of future vaccines and therapeutic candidates.

## Introduction

Chikungunya virus (CHIKV) was first isolated in Tanzania in 1953 [1], and has recently emerged in islands of the Indian Ocean in 2005, and engendered the largest Chikungunya fever epidemic on record [2]. The most affected region was the island of La Réunion, where CHIKV infected approximately a third of the island's inhabitants (i.e., ∼300,000) [3–5]. The outbreak, which now also involves India with an estimated 1.3 million cases [6–8], has a significant potential to spread globally given the wide distribution of its arthropod vector [9,10].

CHIKV is a member of the genus *Alphavirus* in the family of *Togaviridae*. Alphaviruses are small, enveloped viruses with a message-sense RNA genome that encodes four non-structural proteins (nsP1–4) and three structural proteins (C, E1–2). This arbovirus is maintained in nature by uninterrupted cycles of transmission between mosquitoes and vertebrate hosts such as macaques [11–13]. Several alphaviruses cause disease in humans, primarily as a result of epizootic infections. These include the American encephalitic alphaviruses and several species in the Semliki Forest Virus group, principally the Afro-Asian CHIKV, the African O'Nyong-Nyong virus, as well as the Australasian Barmah Forest virus and Ross River virus [14]. CHIKV infection is characterized by fever, arthralgia, myalgia, rash and headache. During the La Réunion Island outbreak, previously unreported severe forms of Chikungunya infection were observed in adults, complicated by encephalopathy and hemorrhagic fever. These severe cases almost exclusively occurred in adults with underlying conditions such as diabetes, alcoholic hepatopathy or impaired renal function [3,15]. Moreover, while CHIKV-associated fatalities had not been reported prior to this outbreak, at least 213 persons infected with CHIKV died in La Réunion Island [16,17]. Finally, although never described before, *per-partum* mother-to-child CHIKV transmission has been observed and is associated with severe neonatal disease characterized in more than half of the cases by encephalopathy [18,19].

The pathophysiology of human CHIKV infection has so far remained essentially unknown, in part because of the lack of a permissive small animal model. In order to gain a better understanding of CHIKV-associated pathophysiology, we have developed a mouse model of infection. Using this model system and comparing it to human samples, we have uncovered the following pathophysiological features of CHIKV infection: (i) viral dissemination and disease severity are strongly increased during the neonatal period, (ii) type-I IFN signalling plays a critical role in the control of the infection and is associated with severe infection when deficient, (iii) symptomatic organs are those infected by CHIKV, and (iv) fibroblasts of connective tissues are prominent cell targets in vivo*,* in permissive mice as well as in humans. Together, this study offers the first in depth in vivo analysis of CHIKV cellular tropism and offers a validated small animal model that may prove useful for the development and testing of novel vaccine and therapeutic strategies.

## Results

### Neonatal Mice Are Permissive to CHIKV Infection

**Figure 1:**
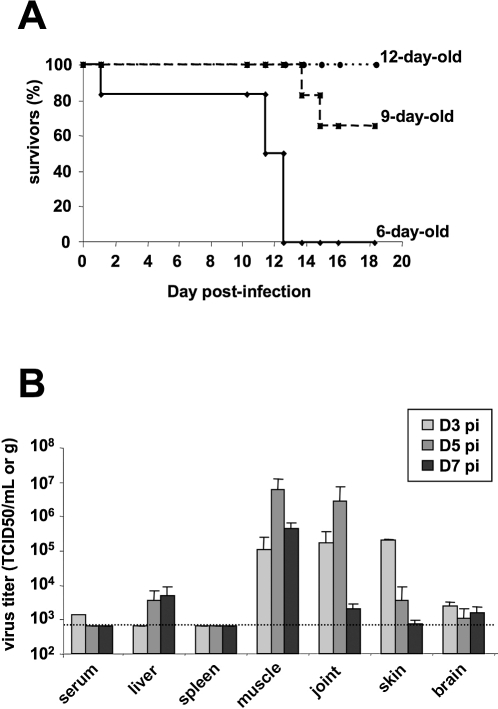
CHIKV Infection in Mouse Neonates (A) Survival of mouse neonates according to their age. Mouse neonates were inoculated with 10^6^ PFU of CHIKV via the ID route and observed for lethality (*n* = 6 per group). (B) Viral titers in tissues of 9-day-old neonates. Mice were inoculated with 10^6^ PFU of CHIKV via the ID route and sacrificed at the indicated time points. The amount of infectious virus in serum and tissues were quantified by TCID50. Each data point represents the arithmetic mean ± SD for at least four mice. The broken line indicates the detection threshold.

In order to study the pathophysiology of CHIKV infection in the adult and neonatal hosts, we aimed to develop a small animal model of infection. We first inoculated a series of classical laboratory mouse strains: outbred OF1 mice and inbred C57BL/6 and 129s/v mice. Intra-dermal (ID) injection of 10^6^ PFU of CHIKV-21, isolated from an individual from La Reunion with central nervous system (CNS) symptoms [4], showed that WT adult OF1 C57BL/6 and 129s/v mice are resistant to CHIKV infection. Neither morbidity nor mortality was observed following infection and no infectious virus could be recovered from tissues (unpublished data). In contrast, neonatal C57BL/6 mice exhibited an age-dependent lethality to CHIKV infection (Figure 1A): six-day-old and 9-day-old mice all developed flaccid paralysis on D6 or D7 post infection (pi), and all 6-day-old mice died before D12 pi, whereas more than half of 9-day-old infected mice recovered. Strikingly, by day 12 of life, C57BL/6 mice showed neither morbidity nor mortality following infection. We investigated the kinetic of virus replication in tissues of 9-day-old mice at D3 and D5 pi and at the onset of symptoms (D7 pi). Infectious virus was detected at low level at D3 pi in serum and at D5 and D7 pi in liver (Figure 1B). Strikingly, at all time points analyzed, infectious CHIKV was detected very abundantly in muscle, joint and skin and to a lower extent in the brain (Figure 1B).

Together, these results establish that, as observed in humans, CHIKV pathogenicity is strongly age-dependent in mice, and that in less-than 12 day-old mouse neonates, CHIKV induces a severe disease. Of note, in the infected neonatal mice, CHIKV is abundantly detected in the same organs than those symptomatic in humans, particularly infected neonates and babies under the age of one year [18].

### IFN-α/βR^+/−^ and IFN-α/βR^−/−^ Animals Provide In Vivo Models for Mild and Severe Forms of CHIKV Infection, Respectively

That neonatal mice but not adult mice were permissive to CHIKV infection ruled out the existence of an intractable species barrier in the mouse. It also questioned the nature of mouse adult non-permissiveness. Guided by the well-established ability of CHIKV and other alphaviruses to trigger type-I IFN synthesis and their sensitivity to type-I IFN responses [20–24], we tested the permissiveness of adult IFN-α/βR knockout (IFN-α/βR^−/−^) mice towards CHIKV.

**Figure 2:**
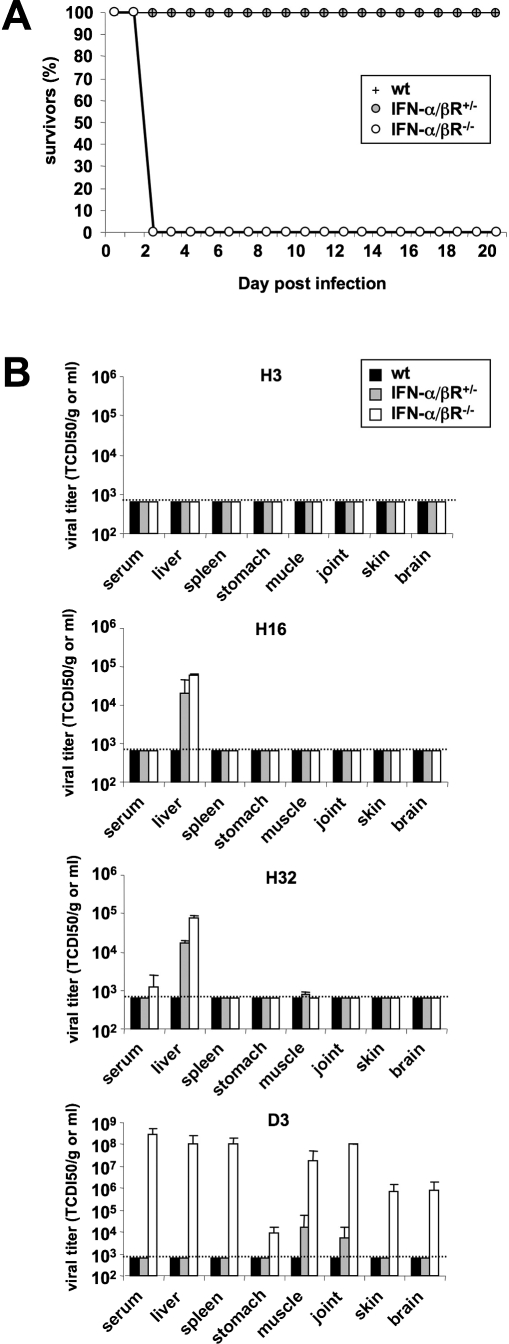
CHIKV Infection in Mice According to the Integrity of the IFNα/β Pathway (A) Survival of mice inoculated with 10^6^ PFU of CHIKV via the ID route (*n* = 6 for each category). (B) Viral titers in IFN-α/βR^−/−^, IFN-α/βR^+/−^, and WT adult mice infected via the ID route with 20, 10^6^, and 10^6^ PFU, respectively. At the indicated time points, mice were sacrificed and the amount of infectious virus present in serum and tissues was quantified by TCID50. Each data point represents the arithmetic mean ± SD for at least four mice. A broken line indicates the detection threshold.

In contrast to WT adult mice, all infected IFN-α/βR^−/−^ adult mice developed a severe disease characterized by muscle weakness of the limbs (i.e., loss of muscle tone) and lethargy and died at D3 pi (Figure 2A). Whereas no mortality was observed in WT adult mice inoculated with 10^6^ PFU of CHIKV-21, the lethal dose 50 (LD_50_) was of 3 PFU in adult IFN-α/βR^−/−^ adult mice, with an average survival of 3 ± 0.2 days. Similar results were obtained when infecting IFN-α/βR^−/−^ adult mice with two other isolates from La Réunion (CHIKV-27 and −115) and one African isolate from Congo (CHIKV-117) (unpublished data). Together, these results indicated that the basis for the resistance of WT adult mice to CHIKV is linked to type-I IFN signalling, which thus stands as a key player in the control of CHIKV infection.

We next investigated CHIKV replication in tissues of WT and IFN-α/βR^−/−^ adult mice inoculated via the ID route. As IFNAR-α/βR^−/−^ adult mice are highly susceptible to CHIKV infection, we inoculated them with about 10 LD_50_, i.e., 20 PFU, whereas we inoculated WT mice with 10^6^ PFU, and determined the viral load in mouse tissues at different time points pi. CHIKV was not isolated from tissues of WT mice at H3, H16**,** H32, D3 pi, or D6 pi (Figure 2B and unpublished data). In IFN-α/βR^−/−^ mice at H16 pi, infectious virus was detected only in the liver. At D3 pi, it was abundantly detected in muscles, joints, skin, and brain with high viral titers also present in serum, liver and spleen (Figure 2B). Similar results were obtained with the Congolese isolate CHIKV-117 (unpublished data).

In search for a model of non-lethal CHIKV infection that would reflect the mild disease predominantly observed in human adults, we evaluated disease pathogenesis in IFN-α/βR^+/−^ adult mice. In IFN-α/βR^+/−^ adult mice –which express IFN-α/βR to a similar level found in WT animals, as assessed by FACS analysis (unpublished data)–, no mortality nor lethargy was observed upon infection with 10^6^ PFU of CHIK-21 (Figure 2A). However, in contrast to what was observed in WT adult mice, infectious virus was recovered from liver as early as H16, at titers similar to that of IFN-α/βR^−/−^ infected mice (Figure 2B). Strikingly, at D3 pi, infectious virus was confined to muscles and joints (Figure 2B) after which the virus was cleared and reached undetectable levels by D6 pi (unpublished data). These data suggest that a dose effect of type-I IFN receptor gene controls the level of permissiveness of adult mice towards CHIKV, with IFN-α/βR^+/−^ and IFN-α/βR^−/−^ adult mice developing a mild and severe infection, respectively. As observed in human clinical cases, the mild form of the disease corresponds to a peripheral infection targeting predominantly skeletal muscles and joints, whereas the severe form is also associated with viral dissemination to other organs, including the CNS.

### CHIKV Cell Tropism in Mice

We next investigated the cell tropism of CHIKV in liver at H16 pi and in peripheral infected tissues, namely, liver, skeletal muscles, joints and skin at D3 pi. In liver of infected adult IFN-α/βR^−/−^ mice at H16 pi, a weak labelling of CHIKV antigens was detected in sinusoidal capillary endothelial cells (Figure S1A–S1C), as well as in some F4/80 labelled mature macrophages (Figure S1D–S1F), whereas at D3 pi, consistent with a sharp increase in liver and serum viral loads, CHIKV immunolabeling was more intense and diffuse, and co-localized within and around sinusoid capillaries (Figure S1G–S1I). Confirmatory findings were obtained by transmission electron microscopy analysis of sections from the same infected livers. These images reveal numerous CHIKV particles budding at the surface of sinusoid capillary endothelial cells (Figure S2A). A lower number of viral particles were also found associated with (arrow) or budding at the surface (arrowhead) of Kupffer cells (Figure S2B). In order to further investigate viral replication in this cell type, we isolated primary liver macrophages and infected them in vitro. Although only low levels of viral replication were observed, we do conclude that liver macrophages are capable of being infected by CHIKV (Figure S1J). In contrast, brain-derived microglial cells were refractory to infection suggesting that macrophages are not a general target of infection (Figure S1J). In spleen, the immunolabelling for viral antigen was exclusively observed in the red pulp, notably in F4/80-positive cells (unpublished data).

**Figure 3:**
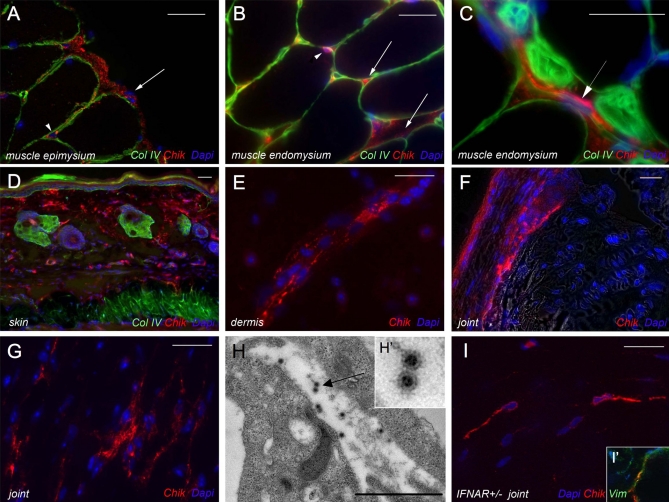
CHIKV Cell and Tissue Tropisms in Mice IFN-α/βR^−/−^ mice ID inoculated with 20 PFU and IFN-α/βR^+/−^ ID inoculated with 10^6^ PFU route were sacrificed at D3 pi. Multiple immunostaining were performed on tissue cryosections from IFN-α/βR^−/−^ mice (A to G) and from IFN-α/βR^+/−^ mouse (I), and transmission electron microscopy on joint from IFN-α/βR^−/−^ mice (H). Nuclei stained by Hoechst appear in blue, CHIKV in red (A to G and I). Basal lamina (collagen IV) (A to D) and mesenchymal cells (vimentin) (I′) appear in green. In all these pictures bar is 10 μm. Muscle fibroblasts, identified notably as cells not surrounded by a basal lamina, display a strong immunostaining for CHIKV in the epimysium (arrow) and endomysium (arrowhead) of skeletal muscle (A). Fibroblasts (arrows) and very few satellite cells (arrowhead) easily recognizable as a single mononucleated cell located beneath the muscle fiber basal lamina are labeled for CHIKV antigens in endomysium of skeletal muscle (B and C). Fibroblasts were also immunostained for CHIKV in deep dermis (D and E) and in joint capsule (F and G). (H) Transmission electron microcopy view of viral particles (arrow in H and H′) in contact and within the cytoplasm of a cell in connective tissue, identified as a fibroblast because of the absence of surrounding basal lamina and the adjacent type I collagen fibers. Infected cells positive for CHIKV in skeletal muscle of IFN-α/βR^+/−^ mouse (I), identified as fibroblasts since they were not surrounded by basal lamina and expressed vimentin (I′).

In skeletal muscles of infected adult IFN-α/βR^−/−^ mice, and to a lower intensity of IFN-α/βR^+/−^ mice, CHIKV immunolabelling was predominantly observed in connective tissue, particularly in the external region, the epimysium (also called muscle fascia), and to a lower extent in the perimysium and endomysium (Figure 3A–3C). Consistent with these observations, viral load was higher in the epimysium than in the perimysium/endomysium (unpublished data). The main target cells in muscles were fibroblasts, as shown by the morphology of the cells and their co-immunolabelling with anti-vimentin and anti-CHIKV antibodies (Figure S3), and the absence of basal lamina surrounding the immunolabeled cells (Figure 3A–3C). Few immunolabeled F4/80-positive macrophages were also observed in the epimysium and to a lesser extent in the perimysium where they predominated around middle size arteries and veins in a close contact with fibroblasts (unpublished data). Consistent with a recent study performed on human material [25], rare satellite cells were also immunolabeled (arrowhead in Figure 3B), readily recognizable as small mononucleated cells located beneath the muscle fiber basal lamina. In skeletal muscles, myofibers were not immunolabelled with anti-CHIKV antibodies.

In IFN-α/βR^−/−^ adult mice at D3 pi, fibroblasts of the joint connective tissue located beneath the synovial wall were also infected (Figure 3F–3H), but both deep articular and osseous tissues (i.e., chondrocytes and osteocytes and osteoblasts) were uninfected. In the skin, viral antigens were also observed in fibroblasts of the deep dermis (Figure 3D, 3E). In IFN-α/βR^+/−^ adult mice at D3 pi, viral antigens were also detected in fibroblasts of the connective tissue of joints (Figure 3I) and skin (unpublished data) but to a lower level than in IFN-α/βR^−/−^ mice.

**Figure 4:**
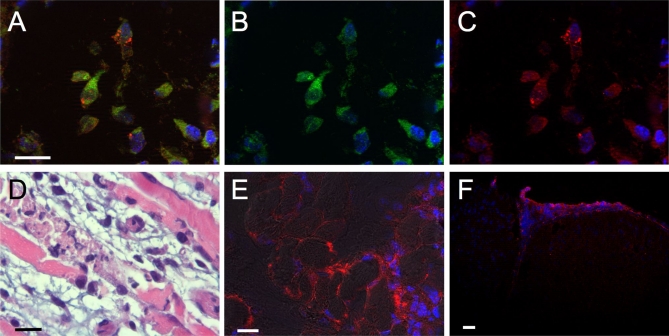
CHIKV Cell and Tissue Tropisms in Mouse Neonates Nine-day old IFN-α/βR^+/+^ mice were infected with 10^6^ PFU of CHIKV by ID route. Overlay image (A) of infected muscle connective tissue immunolabeled for vimentin (B) and CHIKV (C). Hematoxylin and eosin staining (D) of longitudinal section of skeletal muscle shows a severe necrotizing myositis with numerous infiltrates and necrosis of the muscle fibers. Immunostaining of CHIKV antigens (red) and nuclei (blue) on muscle (E) and brain (F) sections. Note that the endomysium displays a strong CHIKV immunolabeling, as well as leptomeningeal cells. Bar is 10μm.

Viral cell tropism in infected peripheral tissues of neonates, including muscle, joint and skin was similar to that of adult mice, with a pronounced tropism for fibroblasts (Figure 4A–4C). A notable difference was the presence of severe necrotic myositis consistent with severe myofiber necrosis and inflammation manifested by the infiltration of lymphocytes and monocytes/macrophages (Figure 4D, 4E). Importantly, our in vitro experiments with primary mouse and human muscle fibroblasts confirm the high permissiveness of this cell type towards CHIKV (unpublished data).

To investigate whether blood leukocytes were a target for CHIKV in vivo, peripheral blood mononucleated cells from infected IFN-α/βR^−/−^ mice obtained in the course of the infection (D1 to D3 pi) were double-stained with an anti-CHIKV and a pan-murine haematopoietic cell marker (CD45.2 antibody) and analyzed by flow cytometry. No infected leukocyte was detectable in the blood of infected mice, indicating that blood leukocytes do not represent a significant cell target for CHIKV in vivo (unpublished data), as also reported in the in vitro context [26].

Together, these data show that in infected peripheral tissues of adult IFN-α/βR^+/−^ and IFN-α/βR^−/−^ mice, as well as in WT neonates, fibroblasts constitute a prominent target cell of CHIKV.

### CHIKV Dissemination to the CNS

**Figure 5:**
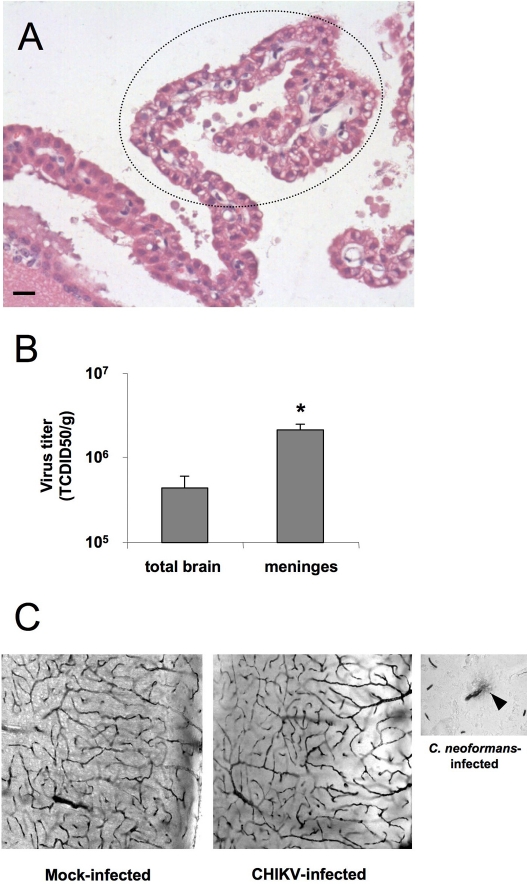
CHIKV Targets the Choroid Plexus and Meningeal Tissue but Does Not Affect the BBB Permeability in IFN-α/βR^−/−^ Mice Mice were infected with 20 PFU of CHIKV via the ID route and sacrificed at D3 pi. (A) Hematoxylin and eosin staining of brain shows a severe vacuolization of epithelial cells of the choroid plexuses (B) The amount of infectious virus present in isolated meninges and in the total brain was quantified by TCID50. Each data point represents the arithmetic mean ± SD for at least three mice (* *p* < 0.05). (C) Representative brain sections of both infected and mock-infected mice after intravenous injection of type VI HRP showing the integrity of the BBB by the absence of any leakage of the staining that remains sequestrated into blood vessels. As a positive control is shown the HRP leakage (arrowhead) induced by the neurotropic yeast Cryptococcus neoformans, which is known to disrupt the BBB integrity.

**Figure 6:**
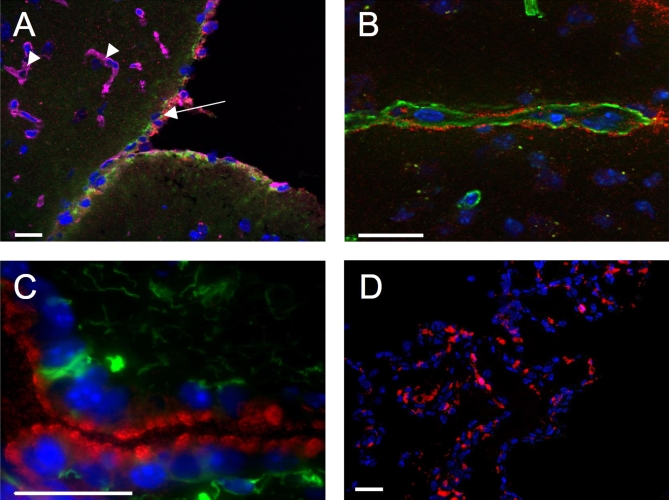
CHIKV Cell and Tissue Tropisms in the Mouse CNS IFN-α/βR^−/−^ mice inoculated via the ID route with 20 PFU were sacrificed at D3 pi and immunostaining was performed on brain cryosections. Nuclei appear in blue and CHIKV in red. Basal lamina (collagen IV) is stained in purple (A) or green (B), and astrocytes and glia limitans (GFAP) appear in green (A and C). Leptomeningeal cells (arrows) display a strong immunolabeling for CHIKV while brain microvessells (arrowheads) and glial cells do not (A). Virchow-Robin spaces showed immunostaining for CHIKV (B) as well as ependymal cells (C) and choroid plexuses (D). Bar is 10 μm.

We next investigated the histopathology and CHIKV infection of the CNS. The only histopathological finding in IFN-α/βR^−/−^ mice at the CNS level was a severe vacuolization of choroid plexus epithelial cells and often of the adjacent ependymocytes (Figure 5A). Choroid plexuses, ependymal wall, and lepto-meningeal cells, including external cells in the Virchow-Robin spaces, were strongly stained for CHIKV, whereas the brain parenchyma did not show significant labelling (Figure 6). We could observe no CHIKV immunolabelling in microglial cells and astrocytes, including those forming the *glia limitans* (unpublished data). The choroid plexuses, which form the blood-cerebrospinal fluid (CSF) barrier, were infected (Figure 6D). In contrast, microvascular endothelial cells that constitute the blood-brain barrier (BBB) were not (Figure 6A, arrowheads). Viral titer in the meninges of infected IFN-α/βR^−/−^ was 5-fold higher than in the total brain (Figure 5B). In the CNS of infected WT mouse neonates, CHIKV infection was also detected at the leptomeningeal level (Figure 4F), but here again, no infection was detected in the brain parenchyma.

To determine whether CHIKV infection altered the permeability of the BBB, we administrated intravenously horseradish peroxydase (HRP), which does not diffuse to the brain parenchyma when the BBB is intact, but leaks into the brain parenchyma in case of BBB disruption [27]. HRP in brains of infected IFN-α/βR^−/−^ adult mice, was confined to the lumen of brain microvessels, as observed in the brains of mock-infected mice (Figure 5C). Thus, despite a strong infection of the meninges and of the Virchow-Robin spaces, the barrier function of the brain microvessels was preserved upon infection.

**Figure 7:**
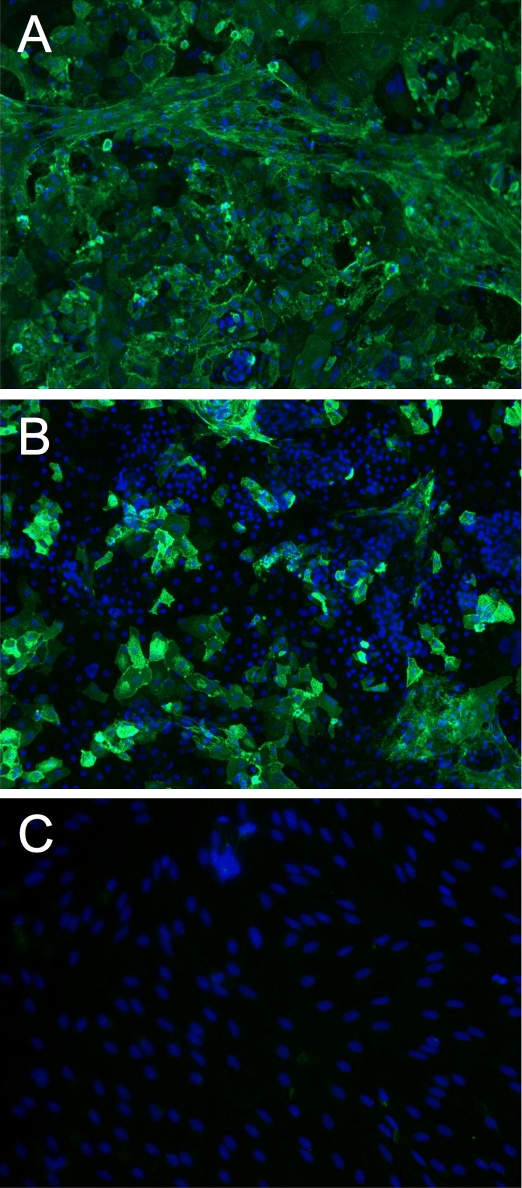
In Vitro Susceptibility of Cells Constituting the BBB Primary cells were infected with CHIKV at a MOI of 10 then viral antigens were detected by immunofluorescence. Apical (A) and basal (B) infection of epithelial cells from choroid plexuses for 18 h. Note the lesser number of immunofluorescent cells following basal infection compared to apical infection. Endothelial cells from brain microvessel were infected for 48 h but viral antigens were not detected (C).

These in vivo findings were confirmed in in vitro BBB systems [28,29]. Primary choroid plexus epithelial cells were highly susceptible to infection via the apical route (Figure 7A) and to a lesser extent via the basal route (Figure 7B), suggesting that CHIKV accesses the cerebrospinal fluid through the choroid plexuses, and may also secondarily infect choroid plexus epithelial cells via their apical surface, thus amplifying viral titers in the CSF. In sharp contrast, primary brain microvessel endothelial cells were fully resistant to CHIKV infection (Figure 7C). Together, these findings suggest that CHIKV gets access to the CNS via the choroid plexuses, and exhibit a marked tropism for the meninges, whereas it does not infect the brain microvessels and parenchyma and does not induce tissue alteration at the brain parenchyma level.

### CHIKV Maternal-Fetal Infection

**Figure 8:**
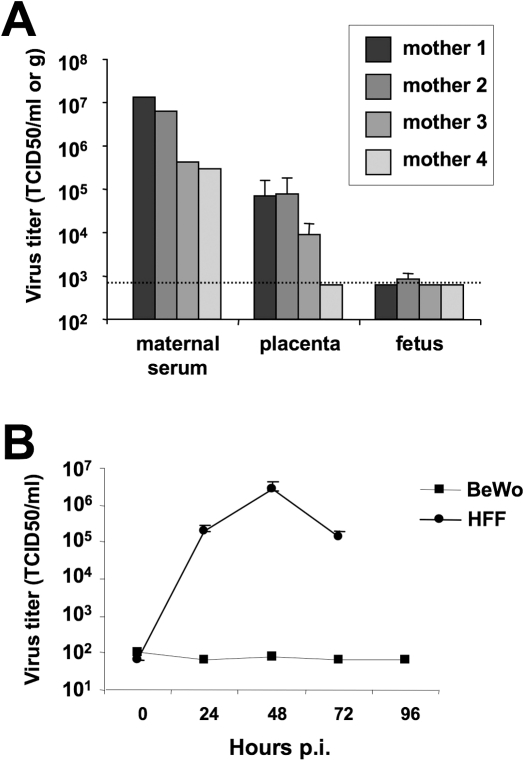
CHIKV Infection in Pregnant IFN-α/βR^−/−^ Mice and Resistance of Human Syncytiotrophoblast Cells to CHIKV (A) Pregnant mice were infected with 20 PFU of CHIKV via the ID route between 16 and 18 d of gestation. Two days later, viral titers were determined in serum and liver, as well as in placentas and fetuses (four mothers, from three to six fetuses per mother). The broken line indicates the detection threshold. (B) The human syncytiototrophoblastic cell line BeWo, and human foreskin fibroblasts HFF as a positive control, were infected with CHIKV at a MOI of 10, and viral load in the culture medium was titrated at the indicated time point by TCID50. Each data point represents the arithmetic mean ± SD for at least four independent experiments.

Given the observations made during the La Réunion outbreak that CHIKV can be vertically transmitted from viremic mothers to their newborns, we investigated maternal-fetal transmission of CHIKV in pregnant IFN-α/βR^−/−^ mice infected with CHIKV-21 via the ID route at D16–18 of gestation. At D2 pi, animals were sacrificed and viral titers determined in maternal serum as well as in placentas and fetuses (Figure 8A). As expected, viral load in maternal serum was elevated. In contrast, placenta viral titers were at least 2 orders of magnitude lower and fetuses were uninfected (Figure 8A). Moreover, no CHIKV immunolabeling could be observed in these placentas (unpublished data). The non-permissiveness of the placental barrier towards CHIKV was confirmed in vitro, by the observation that the human syncytiototrophoblastic cell line BeWo is refractory to infection (Figure 8B).

### CHIKV Tissue and Cell Tropisms in Humans

**Figure 9:**
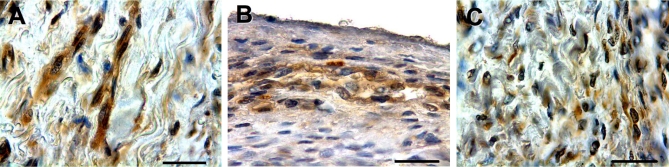
CHIKV Immunolabeling in Human Tissue Samples In human tissues, viral antigens were detected in fibroblasts of dermis (A), of epimysium of skeletal muscle (B) and of joint capsule (C). Bar is 10μm.

In order to test the relevance for human of our in vivo and in vitro studies, we investigated CHIKV cell and tissue tropisms in biopsy samples of CHIKV-infected humans with acute CHIKV infection. We developed a sensitive and specific immunohistochemistry assay (see Materials and Methods) to detect CHIKV antigens in available human tissue samples from a fatal neonatal case. In the tissues that are the classical sites of symptoms in the human disease, namely the skeletal muscles, joints and skin, CHIKV antigens were detected, and viral infection appeared to be confined to fibroblasts of the joint capsule, of skeletal muscle fascia and of the dermis (Figure 9A–9C). As brain human samples were not available, we could not investigate CHIKV dissemination to the CNS. However, studies in experimentally infected *Cynomolgus* monkey, who develop a severe CHIKV infection, indicate that CHIKV disseminates to the CNS, where it targets the choroid plexus and the leptomeninges, but not the brain microvessels and parenchyma (Roques et al. personal communication).

Together, these results demonstrate that in humans, CHIKV is present in symptomatic organs, and that the fibroblast is the privileged cell target in these organs. Moreover, and in agreement with the frequent positivity of CHIKV RT-PCR in the CSF of humans with CNS symptoms [30], CHIKV is able to reach the CNS via the choroid plexuses and preferentially target the leptomeninges in *Cynomolgous* monkey.

## Discussion

Here we have combined in vivo, in vitro, and histopathology approaches to gain a better insight in Chikungunya disease pathophysiology. Using the mouse as a model, we show that in the neonatal host, as well as in adult mice harboring one or two copies of IFN-α/βR null allele, CHIKV exhibits a marked tropism for skeletal muscles, joints and skin, which constitute the classical symptomatic organs in the human disease. This shows that, in contrast to other acute viral infections in which symptoms may predominantly reflect the systemic immune response rather than viral organ dissemination (e.g., influenza), classical symptoms of Chikungunya disease closely reflect CHIKV tissue tropism. Indeed, our study provides direct evidence that in the mouse adult and neonate models, as well as in humans, muscles, joints and skin are privileged CHIKV targets.

We demonstrate here that CHIKV infection severity is critically dependent on two host factors: age and functionality of type-I IFN signaling, thus underlining similarities between CHIKV and the prototypic alphavirus Sindbis [23,31]. In the neonatal host as well as in the adult mouse with a totally abrogated type-I IFN signaling, CHIKV-associated disease is particularly severe, and this severity correlates with higher viral loads and dissemination to the CNS. Importantly, similar findings have been reported in human neonates and adults with severe disease [32]. The reasons why the neonatal status and a defect in type-I IFN signaling favor severe CHIKV infection may partly overlap, but specific neonatal factors may also be involved. Indeed, a number of physiological variables differentiate the neonatal and adult hosts, including the relative proportion of tissue fibroblasts, the rate of cell division, and the maturity and effectiveness of the innate immune system [33]. Future work will have to focus on the similarities and differences between the neonatal and adult hosts with respect to type-I IFN triggering, signaling and responses. Nevertheless, the basic characteristics of CHIKV cell and tissue tropisms are conserved in these two complementary models, and their similarity with what observed in humans strongly argues in favor of their pathophysiological relevance.

With the IFN-α/βR^+/−^ mice, we also provide a model for the benign CHIKV human infection. This animal model should prove very helpful in the development of future vaccine and therapeutic strategies. Importantly, that the gene copy number of IFN-α/βR strictly influences the viral load and tissue distribution as well as the severity of the disease is a strong indication that the strength of type-I IFN signaling likely plays a critical role in the control of CHIKV replication.

The significance of CHIKV specific tissue tropism is emphasized by the observation that tissue fibroblasts constitute the principal CHIKV cell target in all these infected peripheral organs. This in vivo finding is consistent with the in vitro observation that primary mouse muscle fibroblasts are susceptible to CHIKV infection (unpublished data) as well the recent finding by Sourrisseau and colleagues that cultured human lung and mouse skin fibroblasts are permissive to CHIKV [26]. The molecular basis for this prominent in vivo tropism for fibroblasts is unknown and may indicate that fibroblasts could be, relative to other cell types, either (i) in a hyper-permissive status towards CHIKV entry/replication, and/or (ii) in a hypo-sensitive status to type-I IFN-mediated viral interference, making them a target of choice for CHIKV. Interestingly, it is proposed that fibroblasts of connective tissue of dermis, joint capsules [34–36], and muscles have in common the property to form a reticular network of cells interconnected by gap junctions. Whether this characteristic contributes to the selective in vivo hyper-susceptibility of connective fibroblasts to CHIKV infection, and if it plays a role in viral cell-to-cell dissemination deserves future investigations.

Of note, similarly to CHIKV in mouse neonates, Ross River virus, an alphavirus closely related to CHIKV and associated with muscle and joint pathology, has been shown to induce myositis in adult mice [23,37,38]. The absence of myositis in CHIKV-infected IFN-α/βR^−/−^ adult mice could be linked to their early lethality, while it could be linked to the rapid recovery of CHIKV-infected IFN-α/βR^+/−^ adult mice.

Before reaching its target organs, CHIKV undergoes an early burst of viral replication in the liver in IFN-α/βR^+/−^ and IFN-α/βR^−/−^ mice. Indeed, in these mice, the liver is the first and only detectably infected tissue until H32 pi, and CHIKV antigens are primarily detected in sinusoidal capillary endothelial cells and to a lesser extent in Kupffer cells. By D3 pi, and only in IFN-α/βR^−/−^ mice, there is a sharp increase in viremia, with CHIKV antigen detectable in the red pulp of the spleen. This is in contrast with what has been observed with Sindbis virus infection, in which a high level of infection is detected in the spleen of IFN-α/βR^−/−^ mice as early as D1 pi with a subsequent dissemination to the liver at D2 pi [23,37,38]. In the spleen as in the liver, macrophage-dendritic like cells are thought to be the main target cells of Sindbis virus, although by D3 pi in the liver, “sinusoid-lining cells” considered as Kupffer cells and/or endothelial cells are also infected [23]. These in vivo differences between Sindbis (and other alphaviruses such as SFV, EEV or Ross River virus) and CHIKV could reflect the non-permissiveness of dendritic cells to CHIKV (unpublished data and [26]). We also found that, in contrast to mouse liver macrophages, CHIKV is not detected in mouse blood leukocytes in vivo. These data are consistent with previous experiments that showed that human primary monocytes are not permissive to CHIKV, whereas human primary macrophages are [26].

Earlier studies on Sindbis and Ross River also identified the connective tissues of joints and skeletal muscles as sites of viral replication, although the cell type targeted by these viruses in these tissues has not been formally identified [23,37,38]. We show here that this connective tissue tropism also extends to CHIKV. It is thus likely that these shared tropisms and symptoms highlight a common viral pathogenic property, the understanding of which should provide critical clues to the pathophysiologic properties of alphaviruses responsible for arthralgia. Interestingly, both joint and muscle connective tissues contain a high amount of nociceptive nerve-endings [39] that may account for the muscle and joint pain characterizing disease caused by alphaviruses associated with muscle and joint pathology.

In the case of severe CHIKV infection, we found that CHIKV disseminates to the CNS, as also observed in human [30] and non-human primates (Roques et al. personal communication). It is noteworthy that all CHIKV isolates tested exhibited a similar ability to reach the CNS. CHIKV dissemination to the CNS does not correspond to non-specific spreading due to an overwhelming viral multiplication. Indeed, CHIKV is not detected at the brain microvessel and parenchyma levels, but gets access the CNS exclusively via the choroid plexus route, and undergoes a step of viral amplification at the ependyma and leptomeningeal levels. In agreement with these findings, CHIKV is detected in the CSF in humans with severe human Chikungunya disease associated with CNS symptoms [30]. Thus, in contrast to what has been observed for American encephalitic alphaviruses, CHIKV does not appear to be intrinsically encephalitogenic and is associated with reversible CNS symptoms in humans, in line with a virus that does not invade the brain parenchyma nor infect neurons. That CHIKV does not target the brain endothelium and is not detectable at the brain parenchyma level also contrasts with what has been observed with the more closely related Semliki Forest virus, which targets the brain microvessels and also infects neurons [40,41]. Leptomeningeal tissues are, like fibroblasts, of mesenchymatous origin, and exhibits common features with peripheral fibroblastic connective tissue capsules, as they also play a common “envelop” function and form an interconnected multicellular network that acts as a regulatory interface between cerebrospinal fluid and the surface of the brain and between arterioles within the brain and the surrounding neural tissue (Virchow-Robin spaces) [42]. These common tissue organizations and functions may play a role in CHIKV dissemination.

Given its public health and pathophysiological importance, we also investigated CHIKV infection in the pregnant host. By use of the most permissive model we have developed, the IFN-α/βR^−/−^ mice, we show that the placenta does not constitute a privileged target for CHIKV. Indeed, no infected cells can be detected when observing placental tissue sections from infected mice. This is in line with our investigations carried out in human placentas obtained from viremic mothers, in which no infected cell could be detected by mean of immunohistochemistry either [18]. This suggests that viral titers detected in mouse and human placentas rather correspond to a contamination from remaining maternal serum than to an actual placental infection. This is supported by our in vitro finding that human syncytiotrophoblast is refractory to infection, and by the observation that *ante-partum* fetal contamination is exceptional in humans [18]. This provides an explanation for why all cases of vertical transmission of CHIKV in the recent outbreak in La Réunion were observed in *per-partum*, at a time when highly viremic maternal blood can get in contact with the fetal circulation, particularly in the setting of the uterine contractions of the labor, which are known to induce placental barrier breaches. These observations appear to contrast with what observed with Ross River virus, for which actual placental infection and transplacental dissemination have been described both in mice and humans [43,44].

In conclusion, we have developed a mouse animal model for CHIKV infection, allowing us to uncover the viral tissue and cell tropism of this re-emerging alphavirus. We have shown that, in this model, as well as in humans, the fibroblast is the cell type chiefly targeted by CHIKV, and that this accounts for its tropism for muscles, joint and skin connective tissues. The molecular basis for this tropism is currently unknown but may combine specific virus-host cell and tissue interactions as well as an intrinsic relatively lower ability of this cell type to control CHIKV infection. We have also identified two critical factors influencing viral replication, which are the neonatal status, and a defective type-I IFN signaling. Whereas it is clear that an increased neonatal susceptibility is also observed in humans, the relevance of type-I IFN defect as a basis for severe infection in humans remains to be demonstrated. However, the fact that severe infections in humans are exclusively observed in individuals with underlying conditions renders this hypothesis attractive [3,15]. This may indicate that type-I IFN could be of interest to prevent severe disease in adults, as well as in exposed neonates. In addition, the use of neutralizing antibodies also appears interesting, given the strong correlation between viral load and disease severity. A better understanding of the pathophysiology of CHIKV infection and the ensuing development of therapeutic strategies are both critical in the context of a possible globalization of the current CHIKV epidemic.

## Materials and Methods

### Virus.

CHIKV isolates were obtained from individuals during the 2005–06 CHIKV outbreak in La Réunion Island and amplified on mosquito C6/36 cell as described [4]. CHIKV-21 was isolated from the serum of a newborn male with CHIKV-associated encephalopathy; CHIKV-27 was isolated from the CSF of another new-born male with encephalopathy; CHIKV-115 from the serum of a 24-year old female with classical CHIK symptoms. CHIKV-117 was isolated at the Institut de Médecine Tropicale du Service de Santé des Armées (IMTSSA), Marseille, France during the 2000 CHIKV outbreak in Democratic Republic of the Congo from the serum of a person presenting classical CHIK symptoms. Titers of virus stocks were determined by standard Vero cell plaque assay and are expressed as PFU per ml.

### Cells.

Primary choroid plexuses and brain microvascular endothelial cells were obtained, purified and cultured as described [28,29]. The Bewo cell line was obtained from the ATCC. Cells were infected with CHIKV at a multiplicity of infection (MOI) of 10.

### Human tissue.

Human tissue samples were obtained from biopsy specimens collected in the course of the clinical care of people with CHIKV infection in La Réunion.

### Animals.

Outbred OF1 mice, and inbred C57BL/6 and 129s/v mice were obtained from Charles River laboratories (France). IFN-α/βR^−/−^ 129s/v mice were given by F. Tangy with permission from M. Aguet [45]. Mice were bred according to the Institut Pasteur guidelines for animal husbandries and were kept in level-3 isolators. Mice were inoculated by ID in the ventral thorax with 50 μl of a viral suspension diluted with PBS for adult mice and with 30 μl for neonates. Mock-infected mice received PBS alone. Mice were anesthetized with isoflurane (Forene, Abbott Laboratories Ltd, United-Kingdom). Blood was collected by cardiac puncture after which each mouse was perfused via the intracardiac route with 40 ml of PBS at 4 °C before harvesting of organs. Tissues were homogenized, and virus titers of each tissue sample determined on Vero cells by tissue cytopathic infectious dose 50 (TCID50), and viral titers in tissues and in serum were expressed as TCID50/g or TCID50/ml, respectively. The principles of good laboratory animal care were followed all through the experimental process. Pregnant female IFN-α/βR^−/−^ mice at 16–18 days of gestation were infected with 20 PFU of CHIKV via the ID route. At D2 pi, maternal serum, placentas and fetuses were harvested, and viral titers determined in each sample. Mortality studies were performed on groups of six mice and viral titers in tissues from four mice at each time point.

### Histology and immunofluorescence.

Mouse organs were snap frozen in isopentane cooled by liquid nitrogen for cryosectionning or fixed in para-formaldehyde for paraffin embedding. Paraffin-embedded tissues were processed for histological staining (Hematoxylin and eosin). For immunofluorescence, cryosections were fixed for 10 min in ice-cold methanol before incubation for 12 h at 4 °C with the primary antibodies followed by incubation for 1 h with the secondary antibodies. Slides were counterstaining with Hoechst (Vector Lab). The following antibodies were used: polyclonal rabbit anti-collagen IV (Chemicon, Temecula CA, 1:200), polyclonal chicken anti-vimentin (Abcam, Cambridge, UK, 1:200), monoclonal mouse anti-GFAP (BD pharmingen 1:1,000 or 1:5,000 to only see the *glia limitans*), monoclonal rat anti-macrophage antigen F4/80 (Abcam, 1:100), polyclonal rabbit anti-PECAM1/CD31 (Abcam, 1:400), human serum anti-CHIKV were obtained and characterized by the Centre National de Référence des Arbovirus as positive for anti-CHIKV IgM and IgG. The marker specificities were systematically confirmed by examining sections in which the primary antibody was replaced by control isotype or immunoglobulins at the same concentration and by immunostaining of non-infected tissues from the same animal strain. Slides were examined with a Zeiss AxioPlan 2 microscope equipped with an ApoTome system in order to obtain 0.7 μm thick optical sections. Pictures and Z-stacks were obtained using the AxioVision 4.5 software. When necessary, images were processed using the image J software (http://rsb.info.nih.gov/ij/).

### Electron microscopy.

Ultra thins sections were observed at 80 kV accelerating voltage using a JEOL JEM 1010 Electron Microscope.

### Chromogenic immunohistochemistry.

Chromogenic immunohistochemistry was performed on human tissues. After antigen unmasking (97 °C, 20 min), a monoclonal antibody against CHIKV (provided by bioMérieux and the IMTSSA, France) was incubated overnight and revealed with a polymer detection kit (SuperPicTure™ Zymed, http://www.invitrogen.com/) and the DAB chromogen (Vector Lab, http://www.vectorlabs.com/).

### Evaluation of BBB leakage.

BBB permeability was studied using I.V. injection of type VI HRP (Sigma) (30 mg/ml dissolved in Evans blue 2% in 0.9% saline to provide 100 mg/kg) as previously described [27,46]. Five brains were studied for CHIKV-infected mice as well as for mock-infected mice.

### IFN-α/βR expression analysis.

Mouse splenocytes were isolated, labeled with biotinylated goat anti-mouse IFN-α/βR1 antibody (BFA3039, R&D systems), incubated with streptavidin-APC and analyzed by flow cytometry.

## Supporting Information

Figure S1 CHIKV Detection in Liver of IFN-α/βR^−/−^ Mice at H16 pi and D3 pi CHIKV antigens appear in red, CD31 positive cells and F4/80 positive cells in green. At H16 pi, CHIKV antigens were detected in sinusoidal capillary endothelial cells (CD31 positive) (A–C), and in liver macrophages (F4/80 positive) (D–F). At D3 pi, CHIKV antigens were detected within and around sinusoidal capillaries (G–I). (J) Susceptibility of FACS-sorted mouse liver macrophages and microglia to CHIKV. Primary cells were infected with CHIKV at a MOI of 10 then and viral load in the culture medium was titrated at the indicated time point by TCID50. Each data point represents the arithmetic mean ± SD for at least four independent experiments. A broken line indicates the detection threshold.
(3.7 MB TIF)

Figure S2 Transmission Electron Microscopy of Liver Sections from CHIKV-Infected IFN-α/βR^−/−^ Mice at D3 pi On the right pictures are shown enlargement of the boxed regions on the left pictures. Bar is 2 μm on the left and 1 μm on the right. Viral particles are budding (arrowhead) and associated with the plasma membrane (arrow) of sinusoidal capillary endothelial cells (A,A′) and Kupffer cells (B,B′).
(2.6 MB TIF)

Figure S3 CHIKV Target Cells in Muscle are Fibroblasts Immunostaining was performed on skeletal cryosections from CHIKV-infected IFN-α/βR^−/−^ mice. Nuclei were stained by DAPI (blue), CHIKV was detected using a human serum anti-CHIK (red), and mesenchymal cells were stained with rabbit polyclonal antibodies against vimentin (green). Cells of perimysium stained for viral antigens were also labeled with vimentin, indicating they are fibroblasts. Bar is 10 μm.
(1.0 MB TIF)

## Abbreviations

BBB: blood-brain barrier
CNS: central nervous system
CSF: cerebrospinal fluid
D: day
DAB: diaminobenzidine
HRP: horseradish-peroxidase
ID: intra-dermal
IFN-α/βR: IFN-α/β receptor
LD50: lethal dose 50
MOI: multiplicity of infection
pi: post infection
TCID50: tissue cytopathic infectious dose 50
